# Active Ageing, Pensions and Retirement in the UK

**DOI:** 10.1007/s12062-017-9181-7

**Published:** 2017-03-18

**Authors:** Liam Foster

**Affiliations:** Department of Sociological Studies, University of Sheffield, Elmfield Building, Northumberland Road, Sheffield, S10 2TU UK

**Keywords:** Active ageing, Extending working lives, UK, Longevity, Pensions

## Abstract

The ageing population has led to increasing concerns about pensions and their future sustainability. Much of the dominant policy discourse around ageing and pension provision over the last decade has focussed on postponing retirement and prolonging employment. These measures are central to productive notions of ‘active ageing’. Initially the paper briefly sets out the pension developments in the UK. Then it introduces active ageing and active ageing policy, exploring its implications for UK pension provision. It demonstrates that a more comprehensive active ageing framework, which incorporates a life-course perspective, has the potential to assist the UK to respond to the challenges of an ageing population. In doing so it needs to highlight older people as an economic and social resource, and reduce barriers to older people’s participation in society.

## Introduction

The world’s population is ageing. The number of people aged 65 or older worldwide is projected to grow from 524 million in 2010 to nearly 1.5 billion in 2050 (World Health Organisation 2012). The age-based dependency ratio is also changing: in Europe there are four people of working age for every person over 65 and by 2060 there will be only two (European Commission 2010). In the UK it is estimated that the population aged over 65 will grow twice as fast as the working age population, accounting for 24% of the population by 2037 (Office for National Statistics 2015). This process of demographic ageing with the accompanying shifts in the ratio of social security contributors to recipients poses a significant challenge to the sustainability of pensions (Hofäcker 2015).

The ‘active ageing’ framework has emerged as a key policy response to the challenges of an ageing population (Foster and Walker 2015). Active ageing is concerned with enabling people to remain independent and achieve their potential regardless of age. It emphasises the importance of maximizing health, participation and security in enhancing well-being as people age (World Health Organisation 2002). In addition, it indicates the role older people can play in combating the challenges of an ageing population through delaying exit from employment and maintaining an active life following retirement (European Commission 2014). Pension policies can have an important role in extending working lives in addition to providing an adequate income in retirement and, as such, should be a central component of the active ageing agenda (Foster 2012). Despite this, little attention has been given to the links between the active ageing framework and pensions, especially in the UK (Hinrichs and Aleksandrowicz 2006; Botti et al. 2011).

Initially the paper briefly outlines the development of pensions in the UK. Then it considers the emergence of active ageing policy, exploring its implications for UK pensions and extending working lives, before outlining the importance of utilising a life-course approach to active ageing. It shows how an active ageing strategy can assist the UK to respond to the challenges of an ageing population. In doing so it emphasises the need to see older people as an economic and social resource, and reduce barriers to older people’s participation in society.

### The Development of Pensions in the UK

In the UK the fate of the aged poor became a public issue in the late nineteenth century and a campaign for the introduction of an old age pension began towards the end of 1898. This led to the creation of the 1908 Old Age Pensions Act. Coverage was limited for financial and moral reasons, with the focus on undeserved poverty. The level of pension was insufficient to tempt those able to continue in full-time work to stop doing so. Following the Second World War, further changes were made to the state pension scheme based on the Beveridge Report (1942). A flat-rate pension, funded through National Insurance (NI) contributions (made by employees and employers), meant older people became more of a distinct social category, defined by age (of pension entitlement) (Walker 1980). This flat-rate pension was supplemented by an occupational pension for those who could afford them or by national assistance for those without further provision.

These developments enhanced the connection between ageing and public policy in the UK and were accompanied by a rise in living standards of older people (Walker 2009). However they also contributed to the perception of older people as requiring economic support and enhanced ageist stereotypes of old age as a period of dependency and frailty (Townsend 1981). The developments meant older people were expected to exit the labour force at fixed ages and exchange wages for pensions. Pensions operated as a form of institutionalisation of retirement and management of the labour market (Kohli 2007). Retirement provided a clearly defined ‘normative’ phase of the life-course, structurally distinct from paid work, and formed a boundary that produced older people as a distinct group. These developments were underpinned by various types of support created through the welfare state, in addition to the expansion of occupational (defined benefit) pensions (Phillipson et al. 2016).

### Ageing and Neoliberalism

In the 1970s there was substantial change in public discourses on ageing. Policy-makers started to question the cost of population ageing. The rising influence of neoliberalism had a considerable role in these ideological developments. The neoliberal ideology challenged whether the government should provide pensions above a minimum and promoted the role of individual responsibility and markets in providing for individual needs. Retrenchment was justified through a dominant hegemonic narrative of a ‘pension crisis’ in order to enact pension reform along free market principles (Moulaert and Biggs 2012; Macnicol 2015; Grady 2016). Neoliberals asserted that population ageing would have the effect of overwhelming public pension systems, proving unsustainable and creating unfair tax burdens on working age citizens (Foster 2010). For instance, under the Thatcher government ‘sweeping neoliberal claims about unaffordable public spending, and the alleged macro-economic disutilities of public pensions were used to legitimate retrenchment’ (Street and Ginn 2001, p. 42). In 1980, the indexation of state pensions was made less generous and the Social Security Act (1986) modified the Social Security Pensions Act (1975) undermining the redistributive effect of the State Earnings-Related Pension Scheme (SERPS) (an additional state pension for those making particular levels of NI contributions which members of occupational pensions could contract out of). New Labour continued to emphasise the importance of private pension provision but advocated an increased role for means-testing in the form of the Pension Credit, an income related pension top up for those below a specific income. More recently the introduction of auto-enrolment in pensions (discussed later) has further enhanced individual responsibility in relation to pensions (Ginn and Macintyre 2013).

### An Active Ageing Framework

The concept of active ageing, which lacks a precise universally agreed definition, is a relatively new one, achieving widespread currency only in the past 20 years, largely due to the WHO. It emerged at a time when many countries began to restructure their old age income security systems in response to the challenges presented by an ageing population. These changes also fitted with principles of the active ageing framework which ‘portrayed an active role for older people’ (Walker 2009, p. 79). The emergence of active ageing can be traced back to the activity perspective in the US, during the early 1960s, as the antithesis of disengagement, the mutual withdrawal between ageing persons and society (Foster and Walker 2015). Cumming and Henry (1961) assumed disengagement to be universal and inevitable. However, this theory was criticised as it largely ignored older adults’ perceptions about what engagement entailed, enforcing a deficit model (Hochschild 1975). At that time the key to ‘successful ageing’ was perceived as the continuation of activity in older age (Havighurst 1961; Rowe and Kahn 1987). This approach was predicated on reductionist aims and had the effect of placing an unrealistic expectation on individuals in older age to maintain levels of activity associated with middle age, disregarding functional limitations. Thus, it failed to acknowledge the heterogeneous nature of older age. Productive ageing followed in the US a decade later, focussing on ‘any activity by an older individual that produces goods or services, or develops the capacity to produce goods or services’ (Caro et al. 1993, p. 6), before the global concept of active ageing emerged.

Active ageing represents a vision for policy in which facilitating the rights of older people will enable the expanding population to remain healthy (reducing the burden on health and social care systems), stay in employment longer (reducing pension costs), whilst also fully participating in community and political processes. It is concerned with ‘continuing participation in social, economic, cultural, spiritual and civic affairs, not just the ability to be physically active or to participate in the labour force’ (World Health Organisation 2002, p. 12). Therefore, it involves challenging views of older age as characterised by passivity and dependency. It also emphasises the importance of experiences in younger years for determining well-being in later life (Hamblin 2013), highlighting the need for preventative processes throughout the life-course, including providing opportunities to contribute to adequate pension schemes or rewarding periods of caring (Ginn and Macintyre 2013).

Active ageing refutes the ‘decline and loss paradigm’ commonly associated with the consequences of physical decrescence and emphasises the active roles older people occupy in society. It highlighted the need for a distinction between notions of activity and passivity, where being active involves living by one’s own rules as opposed to being ‘normalised’ by others in order to avoid denunciation (Formosa forthcoming). ‘Activity’ should include all meaningful pursuits that contribute to individual well-being such as volunteering or caring roles which should be as valued as paid employment (Foster and Walker 2013). Increased engagement in leisure activities also has the potential to improve health and well-being (Boudiny and Mortelmans 2011). Active ageing also includes the need for activities designed to ensure the protection, dignity and care of older people (Stenner et al. 2011), including a sufficient pension income (Foster 2010). Strategies should be empowering with top-down policy action to facilitate activity, but also opportunities for citizens to take action from the bottom up. Therefore, encouraging older people to be actively engaged with their local communities requires the input of resources. In theory at least, the emergence of active ageing emphasised the need for a departure from notions of ageing in purely economic terms towards a more holistic comprehensive approach which incorporates quality of life, mental and physical well-being and social participation (see Walker 2002, 2009).

There are concerns about how active ageing has been operationalised and that it could end up being counterproductive, oppressive and coercive (Walker 2002; Holstein and Minkler 2007). There is a danger that policy-makers will overemphasise physical activity to the neglect of mental capacity and over-idealise a productive model of active ageing, defining activity according to youthful perspectives which may not be congruent with the experiences of older people (Reed et al. 2003; Barrett and McGoldrick 2013). Thus there needs to be room for alternative lifestyles and definitions of activity (Calasanti et al. 2006). It is necessary to ensure older people are more closely involved in determining what role active ageing could play in their lives (Foster and Walker 2015).

The evolution of a policy discourse on active ageing has been explicitly outlined at a European level and reflected in UK policy. This discourse consists of two contrasting models. Firstly, there is a productivist approach which focuses predominantly on employment policy and the extension of older people’s involvement in the labour market (Lain 2016). In contrast, secondly, there is a more comprehensive approach to active ageing supported by the WHO and United Nations (UN), as well as some parts of the EC (see Walker 2002, 2009 for details). Despite much potential to create the conditions for new, more expansive and positive discourses on ageing the associated emerging policy emphasis has predominantly been on productivity in the form of prolonged employment and pension sustainability (Walker 2009). For instance the EC’s (2009) ‘Ageing Report’ maintained that raising the retirement age, restricting access to early retirement schemes, and a stronger link between pension benefits and pension contributions may create a better incentive to remain in the labour market and the Department for Work and Pensions (DWP) (2014a, p. 5) has argued that ‘early exit from the labour market can have serious implications for the health, well-being and incomes of individuals, and comes at a significant cost to the economy, business and society as a whole’. Moulaert and Biggs (2012) have stated that active ageing has largely come to mean work within the parameters of active ageing policy. There is a danger here that this could result in a new form of ageism, which requires continuation of work and work-like activity as the ‘new legitimacy for a mature identity’ (Taylor and Earl 2016, p. 254).

Productivist active ageing strategies have been justified by the need to raise employment levels, especially given ageing populations and projected pension cost increases. However, in doing so those not in paid employment are excluded from ageing actively, and the valuable contributions they make to society risk being ignored (Boudiny 2013). This productivist and rather utilitarian vision advocates the need for older workers to be ‘activated’ to enhance economic growth. At the same time, in accordance with neoliberal ideologies, an individualisation of the responsibilities of the activated older worker is promoted (Hamblin 2013). This assumes the availability of suitable employment and older people’s capacity to undertake paid work and fails to fully grasp the impact of structural inequities (Depp and Jeste 2006). In practice recent narratives of working longer are imbued with a notion of obligation on the part of the older worker who should have a duty to avoid becoming a ‘burden’ on society (Taylor and Earl 2016). As such the status of being retired is ultimately transformed from a culturally embedded expectation or reward for individuals’ ‘productive’ years to a status more associated with unemployment (Macnicol 2015).

### Pensions and Active Ageing

The fiscal sustainability of pension systems has become firmly entrenched on the policy agenda of the European Union (EU), with the EC (2010) stating that for many countries, including the UK, the future pension situation is untenable. The EU has stated that it will support ‘national pension and retirement reforms that encourage and enable people to earn adequate, sustainable and safe pension entitlements by working longer and increasing their complementary retirement saving in a cost-effective manner’ (European Commission 2012). Productivist notions of active ageing are seen as important mechanisms for enhancing sustainability. In the UK this has led to a focus on pensions and retirement policies. For instance, Ros Altmann (2015, p. 9) the former Pension’s Minister, said:

as people are living longer, we need to rethink what retirement looks like. This is not about forcing people to work on, but supporting those who want to maintain a fuller working life … our concept of retirement and ageing in a workforce must move with the times as people’s lives and the population demographic changes.

This process of ‘rethinking retirement’ has led to a number of employment and pensions policies which are in accordance with productivist aspects of active ageing.

A productivist version of active ageing has focussed on raising the employment rates of older workers through a combination of pension and labour market reforms. Longer working lives are seen as a viable approach to reduce the welfare bill. In 2014 in the UK there were 2.9 million people out of work aged between 50 and the State Pension Age (SPA), the age at which the state pension is received, and the government was spending around £7 billion on out-of-work benefits for people in this age group (Government Office for Science 2016). There are also currently over 1 million people aged 50-SPA who are not working because of sickness or disability (Department for Work and Pensions 2014a). While ‘passive’ programmes tend to discourage the use of early retirement schemes, ‘active’ approaches target employment retention and the reintegration of older workers (Corsi and Samek 2010). Despite considerable increases in older worker’s employment in the UK the current employment rate declines from 86% for 50 year olds, to 65% for 60 year olds and 31% for 65 year olds (Government Office for Science 2016). The DWP ( 2014a) suggests that halving the employment gap between older people aged 50-SPA and those in their 40s could have seen income tax and NI receipts 1% (just under £3 billion) higher and GDP up to 1% (£18 billion) higher in 2013.

Pension system reforms are seen as fundamental to older workers’ employment, by providing effective incentives and an environment for demand and supply of the labour of older workers (Botti et al. 2011). In the UK policy has encouraged delayed retirement in a number of ways, including increases in the age at which the state pension can be received (in 2008, the UK government announced a phased increase in the SPA to 68 by 2046) and gradually standardising the pension age for men and women. These are practices recently undertaken by a number of EU countries (Foster 2012). In an interview with the Guardian Sarah Harper suggested that if Brexit leads to a significant decline in migration in the UK then a reduction in the ratio of workers to retired people is likely to lead to additional increases in the SPA (Lyons and Hill 2017). The changes in SPA may be difficult for people who have already made employment, saving and retirement decisions based on a particular SPA who are unable to adjust to a higher SPA by working or saving longer. Raising the SPA in line with increasing life expectancy may result in more people with health problems or caring commitments leaving the labour market before they are eligible for the state pension (Price et al. 2015). Leaving work before SPA makes it increasingly difficult to maintain living standards into retirement. For instance a third of people who stopped work aged 50 to SPA between 2008 and 2010 saw their overall household income immediately drop by over a half (Department for Work and Pensions 2014a). Weyman et al. (2012) suggest that it is likely to have the greatest effect on middle- to lower-income groups given that they are least likely to be in employment up to SPA. They may be most likely to see the SPA increase as a signal to work slightly longer if possible (Phillipson et al. 2016). For high income groups the state pension represents a relatively marginal factor in their financial planning. They are more likely to have access to occupational pensions and other financial assets.

Caring can have an important impact on employment. The Carers in Employment Task and Finish Group ( 2013) suggest that 315,000 adults below SPA in the UK are out of work, having left work to care for someone. This is most common amongst women as they are more likely than men to undertake caring responsibilities. For instance, in the UK, women spend on average 23 h on family caring, compared with only 10 h by men (Scott and Clery 2012). Children have a considerable impact on women’s employment patterns. In 2010/2012 76% of women aged 21 to 30 without children were working compared to 44% of women with children (Pensions Policy Institute 2016). It is often assumed that family caring means childcare but it includes caring for adults of all ages. In the 50–64 age group, 12% of women and 9% of men were informal carers, mainly for older relatives (Stokes 2013). Unpaid care for parents/in-laws is less visible than childcare, including to employers who may view caring responsibilities for parents as less legitimate than obligations to small children (Loretto and Vickerstaff 2015). In the years approaching SPA, carers’ weekly time spent on eldercare tends to increase, with implications for their pension contributions (Ginn and Macintyre 2013).

Other strategies put in place in the UK to encourage delayed retirement include the option for individuals to defer state pension receipt and increase the final pension amount by 10.4% per year. Until 2005, it was only possible to defer the state pension for up to five years, which resulted in an increased pension amount of 7.4% each year. The UK is also unusual by international standards in significantly raising the SPA without enabling people to take a reduced pension if leaving ahead of SPA (Lain 2016).

The UK Government is increasingly concerned about a widespread lack of adequate saving for retirement. In the UK it has been estimated that approximately 11 million working age individuals will receive lower retirement incomes compared to the level they expect (Department for Work and Pensions 2013). Banks et al. (2005), using 2002–03 English Longitudinal Study of Ageing (ELSA) data for individuals aged between 50 and the SPA, found over 45% of individuals with private pensions overestimated the amount of private pension income they would receive by more than 30% while 20% were unable to give any estimate. Many people do not think about retirement (or believe pension planning is necessary) until they reach their forties (Foster 2017; Bryan and Lloyd 2014). People may also have particular intensions regarding saving for retirement but immediate needs (which may include housing costs and student loan repayments) often mean sacrifices are made to people’s pension saving (Clark et al. 2012). At the same time, there tends to be little awareness of the long-term implications of such decisions (Foster 2017). A lack of pensions knowledge often results in financial decisions being made which are inconsistent with financial needs (Clark et al. 2012). Widespread financial illiteracy means people fail to comprehend pensions and their likely financial position in retirement (Vickerstaff 2010). Decision-making is challenging in a complex pensions environment where individuals, especially women, have unpredictable life-course trajectories (Price et al. 2015).

Ensuring an adequate level of income in retirement for all fits with a more comprehensive approach to active ageing as financial resources can enhance the prospect of ‘activity’ in retirement (Foster and Walker 2015). Government sources of income are crucial to maintaining post-retirement living standards (Phillipson et al. 2016). For instance, in 2012/13 state benefits accounted for 44% of pensioners’ incomes, occupational pensions made up 27%, earnings 17%, investment income 7%, and personal pensions (defined contribution (DC) schemes usually set up by an individual which do not stipulate an employer contribution is required) 4% (Department for Work and Pensions 2014b). Cash benefits/state pensions represented 79% of the incomes of retired households in the bottom quartile in 2012 (Macnicol 2015). In the UK state pension provision is still comparatively low compared with its European counterparts, despite recent changes to the state pension scheme (Creighton 2014). The indexation of the Basic State Pension (BSP), a regular payment from the government that begins when people reach the SPA based on an individual’s history of payments of NI Contributions, and the means-tested Pension Credit has been improved and a new single tier pension, combining the basic and second (earnings-related) state pensions, was introduced in 2016 at about £155 per week if payable in full. Since no extra money is forthcoming, there will be both gainers and losers, with estimates of gainers varying from 35% of men and 61% of women (Crawford et al. 2013) to 70% of men and 75% of women (Department for Work and Pensions 2015). Men and women already over the SPA in April 2016 are ineligible and will continue to receive the state pension in its current form, even where they would have benefitted from the new pension. As such many pensioners, including the oldest old, are excluded from this policy. If existing pensioners had been included, by 2025 this would reduce the projected percentage of pensioners living in relative poverty from around 11% under current policy to around 7%, rather than 10% when just including those reaching the SPA post-2016 (Carrera et al. 2012). Lloyd (2015), using ELSA, found that increasing income in retirement impacts upon retirement satisfaction. Therefore, enhancing the basic level of pension provision could assist older people to finance additional activities, key characteristics of comprehensive active ageing (ILC–UK 2014). Hence the need for a more inclusive approach to basic pension provision which does not exclude the oldest old (Walker 2002).

Over recent years the rise of an anti-collectivist ideology in conjunction with a neoliberal political economy has driven the promotion of private pensions through an emphasis on ‘personal responsibility’. In the UK the move towards DC schemes from Defined Benefit (DB) schemes have accelerated in private pensions, a common trend in European countries. For instance in the UK the number of workers in private sector DB schemes still open to new members is merely 1 million while the total private sector workforce is 23 million (McClymont and Tarrant 2016). DC schemes, unlike DB ones, offer no guaranteed income. Instead, retirement income depends on the performance of the funds invested. The move towards DC schemes represents a change from more buffered and collective private pensions to a more individualised exposure to financial market risks. The guaranteed income provided in DB schemes has led to employees in DB schemes being more likely to retire before the SPA than members of DC schemes (Phillipson and Smith 2006). Furthermore, taxation and the inflexibility of occupational pension rules have been a barrier to individuals taking flexible or gradual retirement. Until 2006 tax rules inhibited taking paid work while also drawing a pension from the same employer (Vickerstaff et al. 2006). An early retirement deterrent was imposed in 2010 with the age threshold for drawing a private pension increased from 50 to 55 years in accordance with productivist active ageing measures (Principi et al. 2016).

Concerns about pension under-provision led to new legislation being implemented in 2012 regarding auto-enrolment into pensions with the National Employment Saving Trust (NEST) as the default option. This has similarities with schemes already introduced in some countries such as New Zealand. It has been argued that this emerged as a result of limited success in assisting people to become ‘rational actors’ in their approach to pension saving by enhancing pension education, improving financial literacy and facilitating choice (see Clark et al. 2012). This reform, targeted at low and medium earners, is intended to offer access to a portable DC occupational pension to the millions of people without access to good quality workplace pension provision while allowing existing employer schemes with benefits or contributions above the NEST’s minimum to continue. Contributions into NEST, or an alternative auto-enrolment scheme, are currently set at 4% for the employee, 3% for the employer and 1% in tax relief (total 8%). It is estimated that around 11 million people will be eligible, with six to nine million people newly saving (Department for Work and Pensions 2013). Less than one in ten has exercised their right to opt out. The logic behind auto-enrolment is that while structured advice and information can improve understanding, behavioural barriers, including myopia, cynicism and inertia, may still stymie increases in saving (Wicks and Horack 2009). It is worth noting that those with an income below GBP £10,000 per annum are not auto-enrolled, and even if those earning below GBP £5824 opt in they will not attract an employer’s contribution (these thresholds are reviewed annually). There are also concerns that the 8% contribution rate is insufficient. As such Grady (2016) argues that the likely outcome of auto-enrolment will be an increased working life for many as recipients will be unable to adequately supplement their work income with their pension. In this context non-retirement may be representative of coercion rather than personal choice.

### Extending Working Lives

In 2010 the default retirement age (DRA) of 65, which meant that employers could force their employees to retire at the age of 65, was abolished in the UK following a number of countries including Australia, Canada, New Zealand and the USA (certain employers such as the fire service still have a compulsory retirement age). The abolition of the DRA in the UK, a productivist active ageing policy, was seen as an important way of ‘encouraging’ people with inadequate retirement incomes to continue working and contributing to pensions (Botti et al. 2011). An active ageing framework needs to acknowledge the diverse experiences and the lack of choice often associated with retirement decisions. The age at which people retire is influenced by a variety of factors including health, wealth, work decisions including involuntary redundancy, caring obligations and job satisfaction (Brown and Vickerstaff 2011). Pension arrangements, and the availability of other benefits (for instance as alternative retirement vehicles), also influence the timing and nature of retirement. Studies regarding the decision to retire find that people with higher incomes and occupational pensions are able to more freely decide when to retire (and tend to be most likely to either retire early or to continue working past SPA), while those without a private pension are more likely to leave work involuntarily, because of redundancy, care-giving responsibilities or ill-health (Phillipson et al. 2016; Vickerstaff et al. 2006). There are also differences between those who ‘consider retirement a well-earned entitlement, individuals who retire for health reasons, those who are compelled to continue working for financial reasons and older workers who have an inherently positive relationship with employment and would like to continue working’ (Beck 2014, p. 201). As such the extending working lives agenda is a complex one, with choice unevenly distributed and constrained by a myriad of interrelated factors (Porcellato et al. 2010; Lain 2016).

Whilst there has been an increase in people working past age 65 in the UK, this largely relates to people being retained in their current jobs rather than undertaking new ones. This is also applicable to self-employed workers where an increase in self-employed individuals aged 65–69 is due to the long-term self-employed remaining in their jobs longer (Phillipson et al. 2016). It may be easier for self-employed workers to work beyond 65 as they are not as confined by organisational rules regarding retirement, have a greater ability to work flexibly and can return to work, having left, without having to convince an employer to rehire them (Lain 2016). Although opportunities to remain in work have improved, it is evident that age discrimination continues in hiring practices (Phillipson et al. 2016). While UK employers often acknowledge the benefits of age diverse workforces, in practice, recruitment trends show they prefer to recruit younger workers (Porcellato et al. 2010; Lain 2016). Stereotypes still exist that older workers: do not want to train; lack creativity; are too cautious; cannot do heavy physical work; and dislike taking orders from younger workers (Walker and Maltby 2012). This is despite evidence which shows older workers are, on average, as effective in their jobs as younger ones (Smeaton et al. 2009). Furthermore, Lain (2012) found that in the early 2000s employees working at age 65–69 who had recently been recruited were disproportionately in low paid, part-time employment which required few qualifications, pointing to the limitations of a productivist active ageing agenda.

The introduction of flexible retirement options has also become a strategy to induce older workers to stay in employment, at least until reaching the official retirement age (Lain 2016). It has been espoused that flexible working, especially part-time work, may offer an attractive way for older workers to stay in work for longer (in accordance with productivist active ageing principles) and could enable them to exercise choice in the timing of their retirement (Loretto and Vickerstaff 2015). For instance, it could take the form of downshifting at the end of their work careers with their current employer and delaying full retirement, or provide ‘bridge jobs’ between the career occupation and full retirement (Vickerstaff 2010). For older workers outside of paid employment access to flexible work arrangements has the potential to facilitate reengagement with the labour market. UK government policy has supported greater flexibility for people to ‘choose a phased approach to retirement’ (Department for Work and Pensions 2006, p. 139) in accordance with the EC (2009) which has also advocated opportunities to extend working lives including flexible working arrangements. In June 2014 the UK introduced the right to request flexible working in the workplace to all employees with at least 26 weeks of service (rather than just parents or carers) with women more likely to request a change than men and more likely to have the request accepted (Smeaton et al. 2014).

Research has shown that there is a strong preference amongst older workers for opportunities to access flexible work options (Smeaton et al. 2009). However, in practice there may be little choice in the decision-making in the context of an increased SPA. Financial security is highly significant in retirement planning, suggesting that whilst it may be a choice to work past retirement, it may represent compulsion for those with inadequate pension coverage (Grady 2016). Furthermore, with the exception of women working part-time (and often on an informal basis to fit with caring responsibilities), relatively few older workers have access to flexible working (Loretto and Vickerstaff 2015). In reality much flexibility in employment is employer-driven and in the form of zero hours contracts and shift systems, and may serve to entrench or worsen poor employment conditions for older workers already disadvantaged by their existing employment circumstances (Taylor and Earl 2016).

While increases in formal retirement ages and the abolition of early retirement opportunities may play a central role in encouraging extending working lives, such policies must not be in isolation from improvements to the quality of work, early intervention to support people with long-term health conditions, and an overhaul of the system of lifelong education and training (having low qualification levels significantly reduces the likelihood of working) (Phillipson et al. 2016). This is a particular problem because older people are competing in the labour market against younger people with more qualifications. Without support people will inevitably find themselves in a position where they are caught between insecure work and an increasingly insecure retirement (Phillipson 2012). In order to address these challenges, and successfully implement a comprehensive active ageing strategy, will require more extensive work by employers to retain and also recruit older workers. Supply-side measures such as raising the SPA, allowing the drawing of pensions whilst still working, and anti-discrimination legislation will all impact on extending working lives but there is an acute need for interventions on the demand-side.

Employers need to recognise that older workers’ lower participation rates in training may, in part, be due to a lack of self-confidence (Porcellato et al. 2010) alongside previous negative experiences with education, especially in classroom-based settings. Currently over 40% of 55 to 64 year olds have undertaken no formal training or education since leaving school (Government Office for Science 2016). In accordance with a comprehensive active ageing strategy, lifelong learning and training throughout the life-course can improve older workers’ qualification levels and their potential ‘employability’ (Walker 2009). Furthermore, the notion of ‘i-deals’, or individually negotiated deals between an employer and employee in connection with decisions around later-life working and retirement, can be important in acknowledging individual choice and heterogeneity of circumstances of employees (Loretto 2016). A holistic i-deal would have many similarities with a comprehensive active ageing approach, recognising options beyond paid employment, including the valuable contributions older people make to society through caring and voluntary work.

### Active Ageing, Pensions and the Life-Course

While active ageing emphasises the need for extending working lives, employment throughout the life-course has considerable implications for pension accumulation. Individuals who have fragmented pension contribution histories are likely to build up smaller pension pots for retirement. Therefore, security in retirement cannot easily be achieved by planning in the immediate run up to retirement (Macnicol 2015). In particular women’s increased risk of poverty in older age depends largely on lower accumulation of pension rights throughout the life-course often as a result of their greater likelihood of undertaking caring responsibilities (Corsi and Samek 2010). British women receive 25% less in state pension than men and women’s private pension savings are only half that of men (Pensions Policy Institute 2016). Such disparities between men and women’s pension income are not specific to the UK. For instance, taking the EU as a whole, men are on average entitled to pensions that are 40% higher than women’s (Tinios et al. 2015).

Given that active ageing policy is dependent on characteristics and experiences throughout the life-course, policies should not simply be targeted at older age. A lack of pension income in later life is a result of insufficient pension saving in early and middle adult life. Therefore, pension challenges will not be addressed only through expanding working life and delaying pension receipt. A comprehensive active ageing approach needs to take into account pension saving throughout the life-course in order to understand income inequalities in older age. This needs to depart from an outdated notion of a ‘standardised’ or ‘normative’ life-course where exit from the labour market in older age is regulated by pension norms (Loretto and Vickerstaff 2015).

While a neoliberal policy approaches may be steering an individual approach to pension saving (Grady 2016), the male breadwinner ideology is still prevalent in pension decision-making and women still undertake greater levels of caring responsibilities at all stages of the life-course (Scott and Clery 2012). Therefore, a comprehensive active ageing strategy needs to recognise the different trajectories women often face and not penalise women (and men) who diverge from traditional male patterns of employment (it is also worth noting that for many men working patterns have changed with more frequent movement between employers and an expansion in self-employment). Unfortunately, neoliberal pension policies, which promote individual responsibility for pension provision, do not account for the realities of many women’s (and some men’s) labour market experience. It would be possible to redevelop pension systems in a manner which de-couples retirement income from labour market participation (Strauss 2014) thus avoiding the penalty for caring years incurred in private pensions (Ginn and Macintyre 2013). In effect, we need to move beyond policy that solely reinforce the liberal celebration of better-paid employment in the labour market and explore ways to reward all forms of work including unpaid labour (Strauss 2014). One option is to introduce an unconditional Citizens Pension set at an adequate level. This approach would reduce gendered inequalities in pensions in retirement and provide a greater acknowledgement of the impact of a life-course approach to active ageing.

## Conclusion

Active ageing has the potential to provide a framework for strategies relating to population ageing and pensions, particularly when a life-course approach is employed. Whilst it is important that a comprehensive active ageing approach recognises diversity and cultural differences, there are fundamental characteristics which must be included in an active ageing approach, whether the focus is on the UK, Europe or beyond. Ultimately it can assist with the process of optimising opportunities for health, participation and security (particularly through increasing the BSP) and as a way to enhance well-being as people age (Corsi and Samek 2010). A comprehensive strategy has the potential to assist with the challenges of workforce ageing and pressure on social protection systems, and enhance the experiences of older people (Foster and Walker 2013). It should not only be concerned with paid employment and pensions; the notion of ‘activity’ should involve all meaningful pursuits that contribute to an individual’s well-being, including their family, local community or society.

In reality, much of the emphasis on active ageing has been on productivity and attempts to encourage people to work longer (Foster and Walker 2015). This focus is likely to adversely affect particular groups, including the lowest paid, carers and those with disabilities, who may have limited opportunities or choice in relation to employment (Phillipson et al. 2016). Furthermore, a greater emphasis on individual responsibility for pension provision has led to the displacement of risk and responsibility from a collective public level to individual policy-holders in the UK and beyond (McClymont and Tarrant 2016). This is likely to present significant challenges to those with limited access to opportunities to save and exacerbate gender inequalities in pension provision (Ginn and Macintyre 2013). In addition, increasing the SPA in the UK may result in more people with health problems or caring commitments leaving the labour market before they are eligible for the state pension (Price et al. 2015).

We need to look beyond narrow productivist notions of active ageing, taking a life-course perspective, to ensure people have the income, opportunities and choices required to enhance their well-being in later life. At the same time, active ageing policies, including economic incentives to continue working in later life, need to be accompanied by policies that enable older workers who want to work to remain active in the paid labour market, focusing on i-deals (Loretto 2016). Unless sustainable pension provision with adequate income in retirement is enacted, there is a danger that more individuals will be compelled to work in insecure forms of paid employment or face an insecure retirement (Phillipson 2012; Grady 2016; Lain 2016). Therefore there is a need to develop a more comprehensive approach to active ageing in the UK which supports retirement as a period of financial security.

## References

[CR1] Altmann R (2015). A new vision for older workers: Retain, retrain, recruit.

[CR2] Banks J, Emmerson C, Oldfield Z, Tetlow G (2005). Prepared for retirement? The adequacy and distribution of retirement resources in England.

[CR3] Barrett G, McGoldrick C (2013). Narratives of (in)active ageing in poor deprived areas of Liverpool. International Journal of Sociology and Social Policy.

[CR4] Beck V (2014). Employers views of learning and training for an ageing workforce. Management Learning.

[CR5] Beveridge W (1942). Report on social insurance and allied services, Cmd404.

[CR6] Botti, F., Corsi, M., & D’Ippoliti, C. (2011). *Active ageing and gender equality: A labour market perspective document de travail working paper No.11–13*. Brussels.

[CR7] Boudiny K (2013). Active ageing: From empty rhetoric to effective policy tool. Ageing and Society.

[CR8] Boudiny K, Mortelmans D (2011). A critical perspective: Towards a broader understanding of ‘active ageing’. Electronic Journal of Applied Psychology.

[CR9] Brown P, Vickerstaff S (2011). Health subjectivities and labour market participation: Pessimism and older workers attitudes and narratives around retirement in the UK. Research on Ageing.

[CR10] Bryan M, Lloyd J (2014). Who saves for retirement? 2: Eligible non-savers.

[CR11] Calasanti T, Slevin K, King M (2006). Ageism and feminism: From ‘et cetera to center. NWSA.

[CR12] Caro F, Bass S, Chen YP (1993). Achieving a productive ageing society.

[CR13] Carrera L, Redwood D, Adams J (2012). An assessment of the government’s options for state pension reform.

[CR14] Clark G, Strauss K, Knox-Hayes J (2012). Saving for retirement: Intention, context and behaviour.

[CR15] Corsi M, Samek L (2010). Active ageing and gender equality policies.

[CR16] Crawford R, Keynes S, Tetlow G (2013). A single-tier pension. What does it really mean?.

[CR17] Creighton, H. (2014). *Europe’s ageing demographically*. ILC-UK 2014 EU Factpack http://www.ilcuk.org.uk/images/uploads/publication-pdfs/Europes_Ageing_Demography.pdf.

[CR18] Cumming E, Henry WE (1961). Growing old.

[CR19] Department for Work and Pensions (2006). A new deal for welfare: Empowering people to work, Cm 6730.

[CR20] Department for Work and Pensions (2013). Supporting automatic enrolment. The government response to the call for evidence on the impact of the annual contribution limit and the transfer restrictions on NEST.

[CR21] Department for Work and Pensions (2014a). *Fuller working lives – A framework for action *https://www.gov.uk/government/uploads/system/uploads/attachment_data/file/458861/fuller-working-lives.pdf.

[CR22] Department for Work and Pensions (2014). The pensioners’ incomes series United Kingdom 2012/13.

[CR23] Department for Work and Pensions (2015). Impact of new state pension on an individual’s pension entitlement –first 15 years.

[CR24] Depp C, Jeste D (2006). Definitions and predictors of successful ageing: A comprehensive review of larger quantitative studies. American Journal of Geriatric Psychiatry.

[CR25] European Commission (2009). Dealing with the impact of an ageing population in the EU (2009 ageing report).

[CR26] European Commission (2010). Private pension schemes: Their role in adequate and sustainable pension.

[CR27] European Commission (2012). Citizen’s summary – White paper on pensions: EU proposals explained.

[CR28] European Commission (2014). Identifying fiscal sustainability challenges in the areas of pension, health care and long-term care polices.

[CR29] Formosa, M. (forthcoming). Responding to the Active Ageing Index: Innovations in active ageing policies in Malta. *Journal of Population Ageing*. doi:10.1007/s12062-016-9163-1.

[CR30] Foster, L. (2010). Towards a new political economy of pensions? The implications for women. *Critical Social Policy, 30*(1), 27–47.

[CR31] Foster, L. (2012). Active ageing and pensions in the EU. *Journal of Comparative Social Welfare, 28*(3), 223–234.

[CR32] Foster, L. (2017). Young people and attitudes towards pension planning. *Social Policy and Society, 16*(1), 65–80.

[CR33] Foster, L., & Walker, A. (2013). Gender and active ageing in Europe. *European Journal of Ageing, 10*(1), 3–10. 10.1007/s10433-013-0261-0 PMC5549231 28804278

[CR34] Foster, L., & Walker, A. (2015). Active and successful ageing: A European policy perspective. *Gerontologist, 55*(1), 83–90. 10.1093/geront/gnu028 PMC4986585 24846882

[CR35] Ginn J, Macintyre K (2013). UK pension reforms: Is gender still an issue?. Social Policy and Society.

[CR36] Government Office for Science (2016). *The future of an ageing population*. http://www.ageing.ox.ac.uk/files/Future_of_Ageing_Report.pdf.

[CR37] Grady J, Manfredi S, Vickers L (2016). Retirement and the pension crisis. Challenges of active ageing for equality law and for the workplace.

[CR38] Hamblin K (2013). Active ageing in the European Union.

[CR39] Havighurst RJ (1961). Successful aging. The Gerontologist.

[CR40] Hinrichs K, Aleksandrowicz P (2006). Reforming European pension systems for active ageing. International Social Science Journal.

[CR41] Hochschild A (1975). Disengagement theory: A critique and proposal. American Sociological Review.

[CR42] Hofäcker D (2015). In line or at odds with active ageing policies? Exploring patterns of retirement preferences in Europe. Ageing and Society.

[CR43] Holstein MB, Minkler M, Bernard M, Scharf T (2007). Critical gerontology: Reflections for the 21st century. Critical perspectives on ageing societies.

[CR44] ILC–UK (2014). Financial wellbeing in later life: Evidence and policy.

[CR45] Kohli M (2007). The institutionalization of the life course: Looking back to looking ahead. Research in Human Development.

[CR46] Lain, D. (2012). Working past 65 in the UK and the USA: Segregation into “Lopaq” occupations? *Work,**Employment & Society, 26*(1), 78–94.

[CR47] Lain D (2016). Reconstructing retirement.

[CR48] Lloyd J (2015). Income, security and wellbeing: Helping savers choose a good retirement.

[CR49] Loretto W, Manfredi S, Vickers L (2016). Extending working lives: What do older employees want?. Challenges of active ageing for equality law and for the workplace.

[CR50] Loretto W, Vickerstaff S (2015). Gender, age and flexible working in later life. Work, Employment and Society.

[CR51] Lyons, K., & Hill, A. (2017). Hard Brexit means retiring later, Britons warned. *The Guardian*: Sunday 15 January.

[CR52] Macnicol J (2015). Neoliberalising old age.

[CR53] McClymont G, Tarrant A (2016). Towards a new pensions settlement: An international perspective.

[CR54] Moulaert T, Biggs S (2012). International and European policy on work and retirement: Reinventing critical perspectives on active ageing and mature subjectivity. Human Relations.

[CR55] Office for National Statistics (2015). Wealth and assets survey 2015.

[CR56] Pensions Policy Institute (2016). The under-pensioned 2016.

[CR57] Phillipson C (2012). Commentary: The future of work and retirement. Human Relations..

[CR58] Phillipson C, Smith A (2006). Extending working life: A review of the research literature.

[CR59] Phillipson C, Vickerstaff S, Lain D (2016). Achieving fuller working lives: Labour market and policy issues in the United Kingdom. Australian Journal of Social Issues.

[CR60] Porcellato L, Carmichael F, Hulme C, Ingham B, Prashar A (2010). Giving older workers a voice: Constraints on the employment of older people in the north west of England. Work, Employment and Society.

[CR61] Price, D., Glaser, K., Ginn, J., & Nicholls, M. (2015). How important are state transfers for reducing poverty rates in later life? *Ageing and Society*, 1–32.

[CR62] Principi, A., Santin, S., Socci, M., Smeaton, D., Cahill, K., Vegeris, S., & Barnes, H. (2016). Retirement plans and active ageing: Perspectives in three countries. *Ageing and Society*.

[CR63] Reed J, Cook G, Childs S, Hall A (2003). Getting old is not for cowards: Comfortable, healthy ageing.

[CR64] Rowe J, Kahn R (1987). Human ageing: Usual and successful. Science.

[CR65] Scott, J., & Clery, E. (2012). *British Social Attitudes 30 - Gender roles*, http://www.bsa.natcen.ac.uk/media/38457/bsa30_gender_roles_final.pdf.

[CR66] Smeaton, D., Vegeris, S., & Sahin-Dimen, M. (2009). *Older workers: Employment preferences, barriers and solutions*. Manchester: Equality and Human Rights Commission. Research Report 43.

[CR67] Smeaton D, Ray K, Knight G (2014). Costs and benefits to business of adopting work life balance working practices: A literature review.

[CR68] Stenner P, McFarquhar T, Bowling A (2011). Older people and ‘active ageing: Subjective aspects of ageing actively. Journal of Health Psychology.

[CR69] Stokes P (2013). Full story. The gender gap in unpaid care provision: Is there an impact on health and economic position?.

[CR70] Strauss K (2014). Accessing pension resources: The right to equality inside and out of the labour market. International Journal of Law in Context.

[CR71] Street D, Ginn J, Ginn J, Street D, Arber S (2001). The demographic debate: The gendered political economy of pensions. Women, work and pensions: International issues and prospects.

[CR72] Taylor P, Earl C (2016). The social construction of retirement and evolving policy discourse of working longer. Journal of Social Policy.

[CR73] The Employers for Carers and Department of Health Task and Finish Group (2013). *Supporting Working carers: The benefits to families, business and the economy. *https://www.gov.uk/government/uploads/system/uploads/attachment_data/file/232302/Supporting_Working_Carers_Summary__accessible_.pdf.

[CR74] Tinios, P., Bettio, F. and Betti, G. in collaboration with Georgiadis, T. (2015). *Men, women and pensions*. Luxembourg: Publication Office of the European Union.

[CR75] Townsend P (1981). The structured dependency of the elderly: The creation of social policy in the twentieth century. Ageing and Society.

[CR76] Vickerstaff S (2010). Older workers ‘the unavoidable obligation’ of extending our working lives. Sociology Compass.

[CR77] Vickerstaff S, Loretto W, White P, Loretto W, Vickerstaff S, White P (2006). The future for older workers: Opportunities and constraints. The future for older workers: New perspectives.

[CR78] Walker A (1980). The social creation of poverty and dependency in old age. Journal of Social Policy.

[CR79] Walker A (2002). A strategy for active ageing. International Social Security Review.

[CR80] Walker A (2009). The emergence and application of active ageing in Europe. Journal of Ageing and Social Policy.

[CR81] Walker A, Maltby T (2012). Active ageing: A strategic policy solution to demographic ageing in the European Union. International Journal of Social Welfare.

[CR82] Weyman A, Wainwright D, O’Hara R, Jones P, Buckingham A (2012). Extending working life: Behaviour change interventions.

[CR83] Wicks R, Horack S (2009). Incentives to save for retirement: Understanding, perceptions and behaviour - a literature review.

[CR84] World Health Organisation (2002). Active ageing: A policy framework.

[CR85] World Health Organisation (2012). *World health statistics*, http://www.who.int/gho/publications/world_health_statistics/2012/en/.

